# KCNQ1 and lymphovascular invasion are key features in a prognostic classifier for stage II and III colon cancer

**DOI:** 10.1186/s12885-022-09473-9

**Published:** 2022-04-08

**Authors:** Sjoerd H. Uil, Veerle M. H. Coupé, Herman Bril, Gerrit A. Meijer, Remond J. A. Fijneman, Hein B. A. C. Stockmann

**Affiliations:** 1 Department of Surgery, Spaarne Gasthuis, Boerhaavelaan 22, 2035 RC Haarlem, The Netherlands; 2 Department of Epidemiology and Data Science, Amsterdam University Medical Center, de Boelelaan 1089a, 1081 HV Amsterdam, The Netherlands; 3 Department of Pathology, Spaarne Gasthuis, Boerhaavelaan 22, 2035 RC Haarlem, The Netherlands; 4 Department of Pathology, The Netherlands Cancer Institute, Plesmanlaan 121, 1066 CX Amsterdam, The Netherlands

**Keywords:** Colon cancer, Prognosis, Biomarker, Classifier, CART, KCNQ1, Lymphovascular invasion

## Abstract

**Background:**

The risk of recurrence after resection of a stage II or III colon cancer, and therefore qualification for adjuvant chemotherapy (ACT), is traditionally based on clinicopathological parameters. However, the parameters used in clinical practice are not able to accurately identify all patients with or without minimal residual disease. Some patients considered ‘low-risk’ do develop recurrence (undertreatment), whilst other patients receiving ACT might not have developed recurrence at all (overtreatment). We previously analysed tumour tissue expression of 28 protein biomarkers that might improve identification of patients at risk of recurrence. In the present study we aimed to build a prognostic classifier based on these 28 biomarkers and clinicopathological parameters.

**Methods:**

Classification and regression tree (CART) analysis was used to build a prognostic classifier based on a well described cohort of 386 patients with stage II and III colon cancer. Separate classifiers were built for patients who were or were not treated with ACT. Routine clinicopathological parameters and tumour tissue immunohistochemistry data were included, available for 28 proteins previously published. Classification trees were pruned until lowest misclassification error was obtained. Survival of the identified subgroups was analysed, and robustness of the selected CART variables was assessed by random forest analysis (1000 trees).

**Results:**

In patients not treated with ACT, prognosis was estimated best based on expression of KCNQ1. Poor disease-free survival (DFS) was observed in those with loss of expression of KCNQ1 (HR = 3.38 (95% CI 2.12 – 5.40); *p* < 0.001). In patients treated with ACT, key prognostic factors were lymphovascular invasion (LVI) and expression of KCNQ1. Patients with LVI showed poorest DFS, whilst patients without LVI and high expression of KCNQ1 showed most favourable survival (HR = 7.50 (95% CI 3.57—15.74); *p* < 0.001). Patients without LVI and loss of expression of KCNQ1 had intermediate survival (HR = 3.91 (95% CI 1.76 – 8.72); *p* = 0.001).

**Conclusion:**

KCNQ1 and LVI were identified as key features in prognostic classifiers for disease-free survival in stage II and III colon cancer patients.

**Supplementary Information:**

The online version contains supplementary material available at 10.1186/s12885-022-09473-9.

## Background

Colorectal cancer (CRC) is one of the most common types of cancer worldwide, with an incidence of nearly 2 million cases annually [1]. A global increase of CRC is foreseen, leading to over one million deaths in 2030. Nevertheless, survival itself has improved due to early detection, better diagnostics and improved treatment over the last years [1–3]. These diagnostic- and treatment strategies nowadays result in 5-years disease-free survival rates of up to 85–90% and 70–75% for stage II and stage III CRC, respectively [3, 4]. However, these are survival rates on a group level, and within both stages survival is different depending on T- and N-status. Furthermore, these survival rates are influenced by the administration of adjuvant chemotherapy (ACT) [5, 6]. All patients with stage III colon cancer have in fact an indication for ACT whereas stage II patients only qualify for ACT in case of high-risk features, which include T4, obstruction, perforation, vascular invasion and harvesting of limited lymph nodes. Besides stage and high-risk features, administration of ACT is subject to patient’s fitness, age and post-operative complications. A reduction of risk of recurrence of 10–16% has been assigned to this use of ACT in colon cancer [7–9]. This effect may be responsible for the relatively high DFS rate of up to 75% [4, 10–13]. However, there is also a subset of patients receiving futile treatment with ACT while suffering its side-effects. Taken together, making decisions who to offer adjuvant treatment based on tumour stage alone has significant limitations and is inadequate.

As many Western countries have implemented CRC-screening programs, a stage-shift is observed, reducing the number of patients who present with advanced cancer whilst increasing the proportion of patients who present with earlier stages of disease. Consequently, the question whether a patient is cured by surgery alone will become increasingly relevant in daily clinical practice, and requires better estimation of an individual’s risk of disease recurrence. Several classifiers have been developed to better identify subgroups of colon cancer patients based on gene expression profiles, like the consensus molecular subtypes (CMS) [14]. However, despite the fact that one of these molecular subtypes was associated with poor prognosis, the diagnostic use of CMS classification has not reached clinical implementation yet.

In daily practice, clinical and pathological features are used to decide which individual patient qualifies for ACT. The pathological features are mainly based on routine immunohistochemical techniques that are widely used. Future prognostic biomarker assays based on immunohistochemistry may therefore be easily implemented in the routine diagnostics process. There is an ongoing quest to discover protein biomarkers that can be evaluated by immunohistochemistry with strong prognostic value, aiming to improve identification of patients with upcoming recurrence, such that ACT may be offered to those most likely to benefit. We previously evaluated tumour tissue expression of 28 proteins and identified multiple biomarkers with prognostic value, such as Aurora kinase A, Lamin A/C, CDX2, KCNQ1 and MACROD2 [15–20].

Despite progress made, it is still not possible to accurately identify all patients with or without upcoming recurrence. Therefore, the need for a prognostic classifier is deemed necessary to tailor treatment strategies in patients with stage II and stage III colon cancer. While we previously analysed many candidate biomarkers individually, the aim of the present study is to analyse what would be the optimal combination of biomarkers to determine prognosis and whether this combination of biomarkers outperforms individual biomarkers and routine diagnostics. Therefore, a prognostic classifier for DFS was built, based on routine clinicopathological parameters and tumour tissue expression data that was available from 28 previously published protein biomarkers.

## Methods

### Study population and clinicopathological features

The study population comprised a well described retrospective cohort of 386 sporadic colon cancer patients. These stage II (*n* = 226) and stage III (*n* = 160) colon cancer patients had their primary surgical resection in the Spaarne Gasthuis (formerly Kennemer Gasthuis) hospital in the Netherlands. The assessment for eligibility for the administration of ACT was based on guidelines available at the time. After surgery specimens were sent to the pathology lab for routine diagnostic workflows and subsequently stored in pathology archives. Clinical data, pathological parameters and archival tumour tissue material were collected in compliance with the ‘Code for Proper Secondary Use of Human Tissue in The Netherlands’ and conform local and national legislation that was applicable at the time [21]. This allowed us to perform the present retrospective observational translational research study without the additional need for study-specific informed consent from individual patients. For 332 patients MSI status was successfully determined previously [22], and was included as clinicopathological parameter. Whole tissue sections were evaluated by a dedicated pathologist for evaluation of LVI, defined as presence of tumour cells within the lumen of lymph vessels, on D2-40 or hematoxylin–eosin stained sections.

### Biomarker features

We previously evaluated 28 protein biomarkers by scoring immunohistochemical stainings of tissue micro arrays (TMA), as described previously [15–20, 23–25]. Details on immunohistochemical staining, scoring, dichotomization and univariate results of these 28 markers are described in Supplementary Table 1. A brief summary of our biomarker workflow is presented in our Supplementary method 1.

### Patient subsets

The cohort contained patients who were not treated with 5FU-based adjuvant chemotherapy (ACT) (*n* = 263) and who were treated with ACT (*n* = 123). Some biomarkers might have prognostic value (risk of disease recurrence) and/or predictive value (responsiveness to ACT). Because it is not possible to distinguish prognostic from predictive impact of biomarkers in patients treated with ACT, CART analyses were performed separately for patients who were and those who were not treated with ACT.

### CART and statistical analysis

**Fig. 1:**
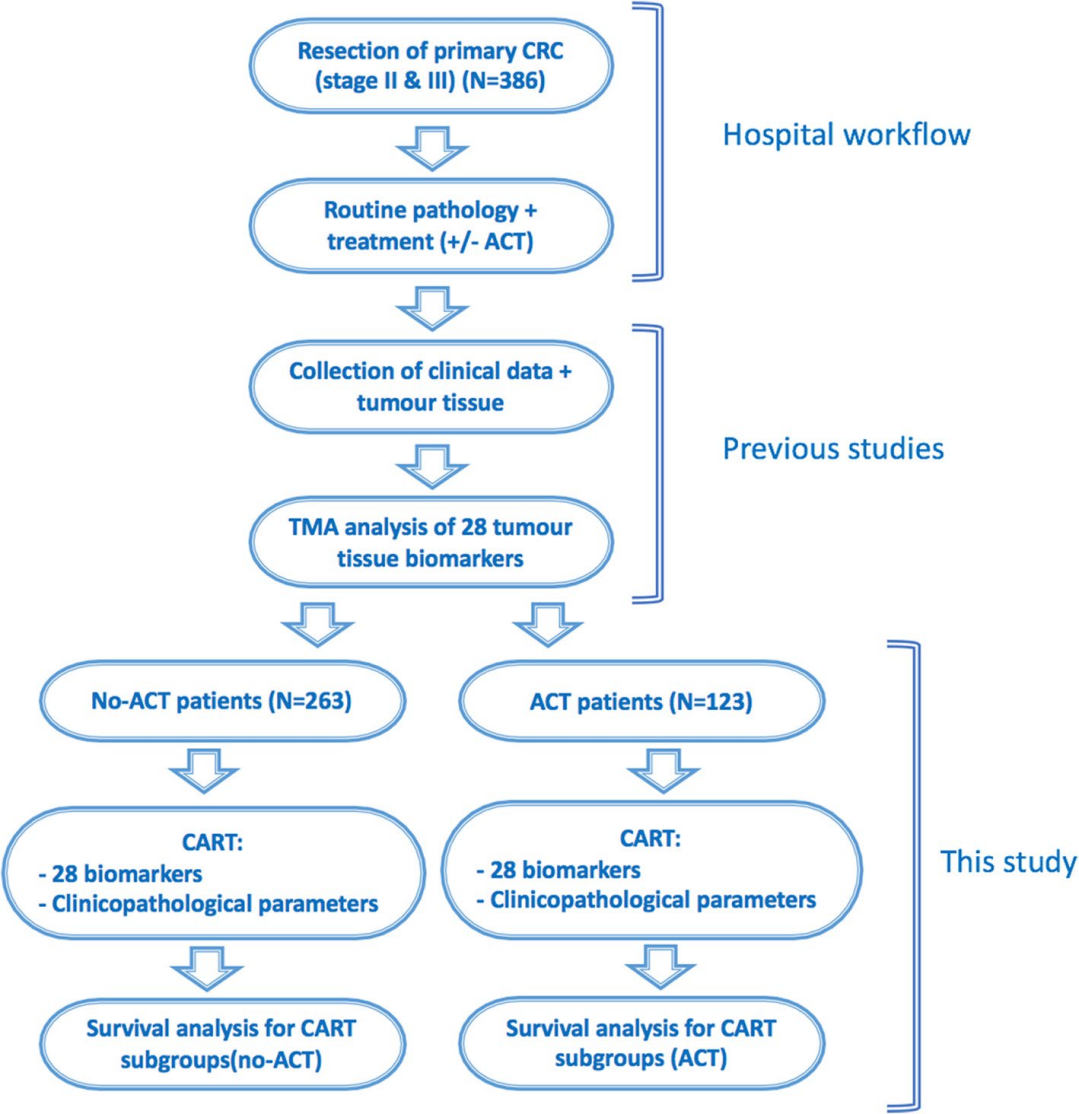
Workflow of this study. All protein biomarker studies on this cohort were based on 386 patients with stage II and III colon cancer. After primary resection, and without disturbing clinical workflows (i.e. diagnostics and the decision of whether or not adjuvant chemotherapy (ACT) was administered), clinical data and tumour tissues were obtained. These tissues were previously analysed for 28 promising biomarkers. These results and routine clinicopathological parameters were included in this CART analysis, with separate analysis for patients not treated and treated with ACT. For each treatment group the classifier was subsequently associated with disease-free survival

Dichotomized data of 28 protein biomarkers were used combined with all available clinical and pathological parameters. Differences in clinical and pathological variables between groups treated with and without ACT were analysed using the chi-square and Mann Whitney U test. For both treatment groups, classification and regression tree (CART) analysis for survival data was performed, aiming to select the best prognostic subset of parameters. CART is a nonparametric approach and therefore does not assume that the data originates from a particular parametric distribution. Furthermore, the CART algorithm incorporates both model fitting and cross-validation to avoid overfitting the model. CART can use the same variables more than once in different parts of the tree: this capability can uncover complex interdependencies or synergies between sets of variables. Endpoint was disease-free survival (DFS), defined as time from surgery to recurrent disease (in months). Minimum number of observations per node was set at 50, and trees were post-pruned by trimming the tree in a bottom-up fashion, until a tree remained with the lowest misclassification error rate [26]. Patients without expression scores for a certain biomarker were allocated based on the ‘logical leaf’, based on distribution of the available expression scores. To assess robustness of the selection of markers in the pruned CARTs, random forests analysis with 1000 trees was performed. Ranking of importance of all markers was obtained. Both CART and Random Forest analysis were performed using RStudio, for which the script is presented in Supplementary Method 2. Subgroups as defined by this CART analysis were used for further statistical evaluation. DFS of these subgroups was visualized by Kaplan–Meier curves, and *p*-values were obtained from log-rank tests. Hazard ratio (HR) with 95% confidence interval (CI) were calculated using Cox regression analysis. Missing values were excluded for survival analysis. Statistical analyses were performed with IBM SPSS Statistics. The workflow of this study was summarized in Fig. 1.

## Results

Of the 386 patients with stage II and III colon cancer included in this cohort, availability of protein expression scores for individual biomarkers ranged from 334 to 384. Median follow up of this cohort was 57,2 months. Recurrence rate was 29.3% and 40.7% (*p* = 0.036) for patients treated without and with ACT, respectively. Furthermore, the group of patients treated with ACT was younger compared to the untreated group (64.9 vs 76.3 year; *p* < 0.001), and included more stage III patients. These, and other baseline clinicopathological parameters included in this CART analysis, stratified for ACT, are shown in Table 1.

**Table 1:** Baseline parameters of this cohort of 386 stage II and III colon cancer patients, stratified for ACT (*P*-values for differences in baseline between ACT treated and untreated patients were calculated using chi-square, or Mann Whitney U when appropriate)

Clinicopathological characteristics	Total: *n* = 386 (%)	No ACT: *n* = 263 (%)	ACT: *n* = 123 (%)	*P*-value
**Sex**				
Male	203 (52.6)	127 (48.3)	76 (61.8)	**0.016**
Female	183 (47.4)	136 (51.7)	47 (38.2)	
**Age,** median (range)(years)	73.1 (28,5 – 94.0)	76.3 (28.5 – 94.0)	64.9 (34.5 – 83.3)	** < 0.001**
**Right sided tumour**	173 (45.1)	117 (44.5)	56 (45.5)	0.91
**Diameter,** median (range)(mm)	40.0 (10.0 – 130.0)	40.0 (10.0 – 130.0)	35.0 (10.0 – 100.0)	**0.009**
**Histological grade**				
Well	24 (6.2)	17 (6.5)	7 (5.7)	0.93
Moderate	302 (78.2)	206 (78.3)	96 (78.0)	
Poor	60 (15.5)	40 (15.2)	20 (16.3)	
**Stage**				
II (= N_0_)	226 (58.5)	192 (73.0)	34 (27.6)	** < 0.001**
III (= N_+_)	160 (41.5)	71 (27.0)	89 (72.4)	
**Tumour stage**				
T1	4 (1.0)	2 (0.8)	2 (1.6)	**0.022**
T2	19 (4.9)	8 (3.0)	11 (8.9)	
T3	325 (84.2)	231 (87.8)	94 (76.4)	
T4	38 (9.8)	22 (8.4)	16 (13.0)	
**Nodal stage (stage III)**				
N1	110 (28.5)	54 (20.5)	56 (45.5)	** < 0.001**
N2	49 (12.7)	17 (6.5)	32 (26.0)	
**Mucinous differentiation**	82 (21.2)	64 (24.3)	18 (14.6)	**0.033**
**Isolated tumour deposits** (ITD)	50 (13.0)	24 (9.1)	26 (21.1)	**0.001**
**MSI-status**				
MSI	65 (16.8)	47 (21.4)	18 (16.1)	0.31
MSS	332 (86.0)	173 (78.6)	94 (83.9)	
**Ulceration**	297 (76.9)	196 (74.5)	101 (82.1)	0.12
**Lymphovascular invasion** (LVI)	78 (20.2)	44 (16.7)	34 (27.6)	**0.015**
**Emergency surgery**	51 (13.2)	35 (13.3)	16 (13.0)	1.0
**Perforation**				
Before surgery	16 (4.1)	12 (4.6)	4 (3.3)	0.38
During surgery	5 (1.3)	4 (1.5)	1 (0.8)	
After surgery	10 (2.7)	9 (3.4)	1 (0.8)	
**Tumour spill**	12 (3.1)	9 (3.4)	3 (2.4)	0.76
**Recurrence**	127 (32.9)	77 (29.3)	50 (40.7)	**0.036**
**CRC mortality**	101 (26.2)	64 (24.3)	37 (30.1)	0.26
**Overall mortality**	177 (45.9)	133 (50.6)	44 (35.8)	**0.008**
**Follow-up** (median, range) (months)	57,2 (3 – 148)	57.1 (3 – 148)	57.5 (3 – 127)	0.73

### CART analysis of patients not treated with ACT

**Fig. 2:**
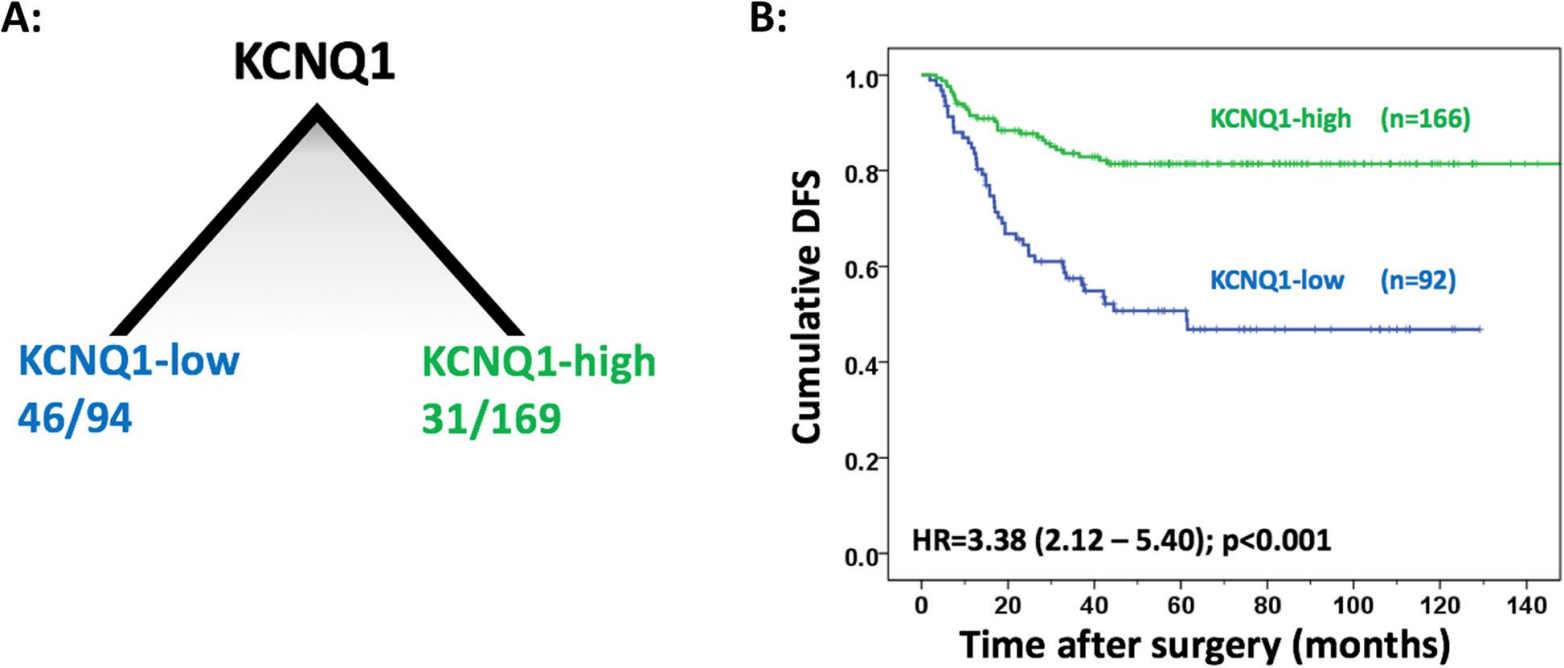
**A** Pruned tree based on CART analysis for patients not treated with ACT, showing two distinct classes based on expression of KCNQ1, with the number of events (recurrence) and the total number of patients in each class. **B** Disease-free survival (DFS) for patients not treated with ACT, visualised by Kaplan Meier curves and stratified for KCNQ1-low and KCNQ1-high. Cox regression HR (95% CI) and *P*-values are reported

For ACT-untreated patients (*n* = 263) the pruned tree with lowest misclassification error rate consisted of one node only, i.e. KCNQ1 (Fig. 2a). The first leaf was defined as KCNQ1-low (*n* = 94) with 46 recurrences (48.9%). The second leaf was defined as KCNQ1-high (*n* = 169) with 31 recurrences (18.3%). Cox regression analysis based on this stratification, showed that the subgroup with loss of expression of KCNQ1 was significantly associated with poor survival (HR = 3.38 (95% CI 2.12 – 5.40); *p* < 0.001; Fig. 2b). The original unpruned tree is shown in Supplementary Figure 1.

### CART analysis of patients treated with ACT

**Fig. 3:**
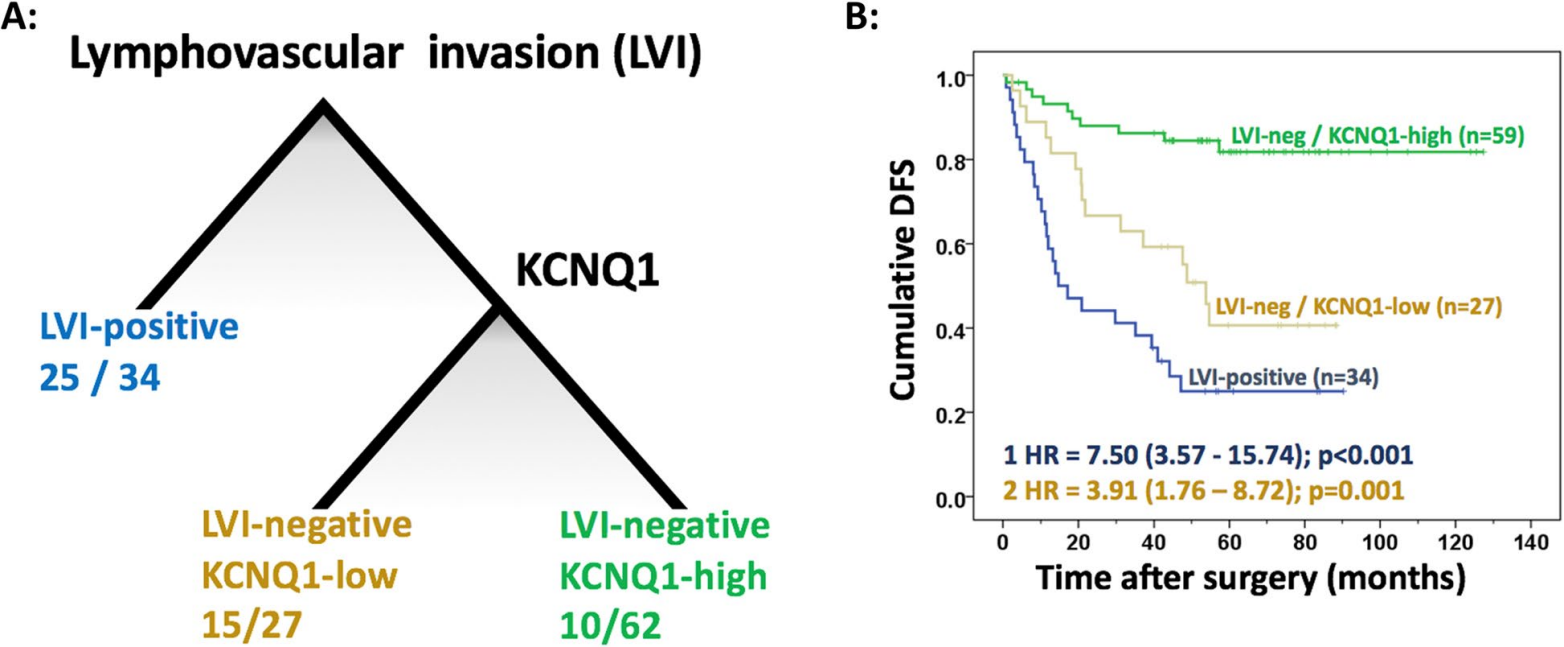
**A** Pruned tree based on CART analysis for ACT-treated patients, showing three distinct classes based on lymphovascular invasion (LVI) and expression of KCNQ1, with the number of events (recurrence) and the total number of patients in each class. **B** Disease-free survival (DFS) for patients treated with ACT, visualised by Kaplan Meier curves and stratified for the three subgroups of the CART analysis. Cox regression HR (95% CI) and *P*-values are reported, with LVI-negative/KCNQ1-high (green) as reference category

For ACT-treated patients (*n* = 123) the pruned tree consisted of 2 nodes, i.e. LVI and KCNQ1 (Fig. 3a). The first leaf consisted of patients with LVI (*n* = 34) with 25 recurrences (73.5%). The second leaf consisted of patients without LVI that were KCNQ1-low (*n* = 27), with 15 recurrences (55.6%). The third consisted of patients without LVI that were KCNQ1-high (*n* = 62), with 10 recurrences (16.1%). Cox regression analysis and Kaplan Meier curves showed that DFS was significantly different between these three subgroups. Patients with LVI showed poorest prognosis, whilst patients without LVI but with high expression of KCNQ1 showed most favourable prognosis (HR = 7.50 (95% CI 3.57—15.74); *p* < 0.001). Patients without LVI and with loss of expression of KCNQ1 had intermediate prognosis compared to the most favorable subgroup (HR = 3.91 (95% CI 1.76 – 8.72); *p* = 0.001; Fig. 3b). The original unpruned tree is shown in Supplementary Figure 2.

### Random forest analysis and variable importance

The ranking of importance of all features is shown in Supplementary Figure 3. Variable importance was defined as the proportion of times a variable is selected in the fitted trees within a random forest, and visualized by variable importance graphs. For the patients not treated with ACT, KCNQ1was the most important feature. For the ACT-treated patients LVI, KCNQ1 and MACROD2 were key features.

## Discussion

In this study, we aimed for a classifier based on a minimal set of complementary markers with maximal prognostic value for stage II and III colon cancer. For patients not treated with ACT KCNQ1 was the single best marker to stratify for, with survival benefit for patients with high expression of KCNQ1. For ACT-treated patients stratification for LVI at first, followed by expression of KCNQ1 for the LVI-negative tumours, was most informative. Tumours with LVI were associated with poor survival. For the subset of tumours without LVI, high expression of KCNQ1 was subsequently associated with the best survival.

The fact that KNCQ1 and LVI were the main and only prognostic features in the pruned CART analyses implies that, following stratification for these features, there were no further CRC subgroups for which any of the other protein biomarkers could provide additional prognostic information. These features were even stronger than tumour stage and MSI-status, which are known to have relatively strong prognostic value and were each included as variables in the CART analysis. Instead, MACROD2 was a feature of similar importance as KCNQ1 and LVI in the group of patients treated with ACT (Sup Fig. 3). Previous analysis showed that MACROD2 was a predictive biomarker for response to ACT in stage III microsatellite stable (MSS) patients [20] and as such its potential relevance in ACT-treated patients was in line with our expectations.

The results of these classifiers emphasize that KCNQ1 was identified as a strong prognostic biomarker for disease recurrence in both stage II and III colon cancer patients, irrespective of MSI-status and/or treatment with ACT [16]. *KCNQ1* encodes an ion channel protein, which acts both as a target gene and regulator of the Wnt/β-catenin pathway [27, 28]. Loss of KCNQ1 is associated with poor prognosis, CRC cell proliferation, epithelial-to-mesenchymal transition and tumorigenesis [18, 27, 28]. In this study, loss of KCNQ1 protein expression appears to be the most informative prognostic feature among 28 promising biomarkers and routine clinicopathological parameters, together with LVI in ACT-treated patients.

LVI is also known as a strong prognostic factor associated with more aggressive tumour behaviour and poor prognosis [29–31]. In theory, all stage III patients are expected to have LVI to some degree because LVI is required to enable lymphatic spreading of tumour cells, and thus progression from stage II to stage III CRC [32]. However, tumours with LVI may not show this feature in every tissue-section that is evaluated by a pathologist, which explains the apparent discrepancy between the amount of stage III (*N* = 160) and LVI-positive (*N* = 78) tumours, at a ratio that is also observed for other patient cohorts [33]. Vascular invasion, and especially extramural vascular invasion, is also associated with poor prognosis, but is less common than LVI and beyond the scope of this study [34, 35].

The present study indicates that determination of KCNQ1 protein expression in patients not treated with ACT, and LVI-status combined with KCNQ1 expression in patients treated with ACT is currently the optimal approach to determine prognosis with a minimal number of key prognostic features. In particular for the group of patients treated with ACT the HRs observed for the combined LVI and KCNQ1 analysis (HR = 3.9 and HR = 7.5; Fig. 3B) exceeded the univariate HRs of LVI (HR = 3.5) and KCNQ1 (HR = 3.3), respectively (data not shown). Whether this is just informative for prognosis, or that it might help the selection of patients who would benefit from ACT, remains to be validated in an independent cohort. Moreover, combining tumour tissue analysis with other techniques to identify high-risk tumours, like measuring post-surgical liquid biopsy cell-free circulating tumour DNA as a marker for minimal residual disease, may further enhance prognostic value of tumour tissue-based classifiers [36, 37].

## Conclusion

KCNQ1 and lymphovascular invasion were identified as key features in classifiers for prognosis in stage II and III colon cancer patients, either not treated or treated with ACT. Although this classifier was not able to create a prediction model for future patients yet, it reinforced the prognostic value of KCNQ1 and lymphovascular invasion, and the need to prospectively evaluate (the combination of) these biomarkers in future studies.

## Supplementary Information

**Additional file 1: Supplementary Table 1.** overvies of the biomarkers previously published based on this cohort and included in the CART analysis.

**Additional file 2.** Supplementary method (biomarker workflow).

**Additional file 3.** Supplementary method (CART script for R-studio).

**Additional file 4: Supplementary figure 1.** Unpruned classification tree by CART analysis for ACT-untreated patients. **Supplementary figure 2.** Unpruned classification tree by CART analysis for ACT-treated patients. **Supplementary figure 3.** Variable importance graphs for both ACT-untreated and -treated patients. LVI = lymphovascular invasion / ITD = isolated tumour deposits / Emergency = emergency surgery / ACT = adjuvant chemotherapy.

## Data Availability

Additional study data is available from the corresponding author on reasonable request.

## Abbreviations

ACT: Adjuvant chemotherapy
CART: Classification and regression tree
CI: Confidence interval
CMS: Consensus molecular subtypes
CRC: Colorectal cancer
DFS: Disease free survival
HR: Hazard ratio
LVI: Lymphovascular invasions
MSI: Microsatellite instable
MSS: Microsatellite stable
