# Supplementary material for: KCNQ1 and lymphovascular invasion are key features in a prognostic classifier for stage II and III colon cancer
Source: BMC Cancer. 2022 Apr 8;22:372. doi: 10.1186/s12885-022-09473-9 (PMC8991490; doi:10.1186/s12885-022-09473-9)
Supplement: Supplementary file 2 — Additional file 2. Supplementary method (biomarker workflow). [file 12885_2022_9473_MOESM2_ESM.docx]

**Supplementary data: brief summary of the biomarker workflow (KCNQ1 example)**

Cohort

The cohort consists of 386 stage II and III colon cancer patients. All patients had surgery for primary colon cancer.

Tissue

Formalin-fixed paraffin-embedded archival tumour tissue was obtained. A total of 6 core biopsies per patient/tumour was punched from the donor blocks and placed into recipient TMA-blocks.

Staining

Sections from these TMA-blocks were cut, 4 µm thick, mounted on glass slides, deparaffinised by xylene and rehydrated with a decreasing alcohol series. Staining for KCNQ1 was performed following antigen retrieval by microwave heating in citric acid (10mM, pH 6.0) and endogenous peroxidase neutralisation in 0.3% hydrogen peroxide in methanol for 25 min, as described previously (Than et al, 2014). The primary rabbit polyclonal antibody directed against human KCNQ1 (sc-20816; Santa Cruz Biotechnology Inc., Santa Cruz, CA, USA) was incubated overnight at a 1 : 200 dilution at 4 ˚C, followed by incubation with anti-rabbit secondary antibodies for 30 min at room temperature (Envision Plus; Dako, Heverlee, Belgium). Secondary antibodies were visualised by liquid diaminobenzidine substrate chromogen system. Slides were counterstained with Mayer’s hematoxylin.

Scoring

All stained TMA sections were digitally captured using the Mirax slide scanner system, and each core was annotated. Scoring templates were made after reviewing the results of the staining process, with examples of negative (no expression at all), weak and moderate, and strong (strongest expression as seen in this batch). For each staining/antibody, the location of the expression to be scored was determined (cytoplasm, nucleus, etc).

Using this template, each TMA tissue core was scored by the first author using Pannoramic viewer, a software program that presented each core, without recognisable patient data. A portion of the cores was independently scored by a second observer as well. Expression scores per core were saved and exported to a database, and linked to the appropriate study number/patient. From each patient, the highest expression score was exported to a database with all clinical and pathological data.

Dichotomization

The highest expression scores were dichotomised for further analysis of patient subgroups using a script for Rstudio. First, the data was randomly split into five subsets. Next, the optimal cut-off for dichotomising scores into a high- or low-expression group was based on four-fifth of the data set using receiver operating characteristic curve analysis for survival data with 5-year DFS as the outcome of interest. This procedure was repeated five times, with one-fifth of the data set varying. The final cut-off was the cut-off that was most often selected. In this way, the optimal cut-off for KCNQ1 was set to ‘low expression’ for negative and weak intensity scores and ‘high expression’ for moderate and strong intensity scores (den Uil et al, BJC, 2016).

Statistics

Differences in baseline characteristics between the groups with high and low expression were analysed. HR, 95% CI, and P-values were calculated using Cox regression analysis. Disease free survival was visualised by Kaplan–Meier curves and compared using the log-rank test. All statistical tests were two-sided. P-values of 0.05 were considered significant. All statistical analyses were performed in SPSS.

Reference

Loss of KCNQ1 expression in stage II and stage III colon cancer patients is a strong prognostic factor for disease recurrence. den Uil et al, Br J Cancer 2016 Jun 12,115(12), 1565-1574).
