# Supplementary material for: KCNQ1 and lymphovascular invasion are key features in a prognostic classifier for stage II and III colon cancer
Source: BMC Cancer. 2022 Apr 8;22:372. doi: 10.1186/s12885-022-09473-9 (PMC8991490; doi:10.1186/s12885-022-09473-9)
Supplement: Supplementary file 3 — Additional file 3. Supplementary method (CART script for R-studio). [file 12885_2022_9473_MOESM3_ESM.docx]

**Supplementary methods: script for CART analysis in R.**

The script for CART and random forest analysis, written for R(Studio). For the use of this script, the parameters in your database should be named exactly the same as in this script, or adjust the names in this script according to your database. All libraries are required, and we chose to import our data (markers and clinicopathological) in a *.Rdata* format (at “markers.df”).

## CART analyses multiple markers ##

### vignette("longintro", package = "rpart")

library(randomForestSRC)

require(randomForestSRC)

library(Hmisc)

require(Hmisc)

library(pROC)

library(rpart)

require(rpart)

library(rpart.utils)

require(rpart.utils)

library(survival)

require(survival)

library(pec)

require(pec)

library(ROCR)

require(ROCR)

library(ipred)

require(ipred)

library(MASS)

library(data.table)

require(data.table)

library(MASS)

require(MASS)

library(rms)

library(ResourceSelection)

library(glmnet)

library(caret)

library(ibb)

library(plotmo)

library(ggplot2)

library(tree)

require(tree)

markers.df <-load("/…/...RData")

#View(markers.df)

#View(biomarker.df)

#View(CART_parameters.df)

setwd("~/Desktop/…/…/output… ")

dim(CART_parameters.df)

hist(biomarker.df$DiseaseFree_survival)

which(is.na(biomarker.df$DiseaseFree_survival))

table(biomarker.df$recurrence)

which(is.na(biomarker.df$recurrence))

table(biomarker.df$KCNQ1)

which(is.na(biomarker.df$KCNQ1))

table(biomarker.df$stage)

table(biomarker.df$MSIstatus)

table(biomarker.df$diff)

table(biomarker.df$mucinous)

table(biomarker.df$Diameter)

hist(biomarker.df$Diameter)

table(biomarker.df$n)

table(biomarker.df$t)

table(biomarker.df$age)

hist(biomarker.df$age)

table(biomarker.df$ACT)

#variable.names(biomarker.df)

vars<-c("sexe","age","emergency","rightsided","t","n","ITD","Diameter","mucinous","diff","ulceration","LVI"," MSIstatus","stage","perforation","spill","ACT","KCNQ1","betaCat","CD44","HTR2B","MACROD2","HIF1a","VEGFA","CDX2","p53","CyclineD1","EGFR_memb","p27","HER2NEU","MIB1","TS","p21","AURK","Bcl2","FAS","FasL","BCLX","BAX","XIAP","cFLIP","Jol2","LUM","VCAN")

regform <- paste(

"Surv(biomarker.df$DiseaseFree_survival, biomarker.df$recurrence) ~",

paste(vars, collapse="+")

)

regform

### CART ########

minsplit_now=50

biomarker.tree <- rpart(as.formula(regform), data = biomarker.df,

method = "exp",

control = rpart.control(minsplit = minsplit_now, cp = 0.01))

biomarker.tree$cptable

cp <- biomarker.tree$cptable[which.min(biomarker.tree$cptable[,"xerror"]),"CP"]

cp

plotcp(biomarker.tree)

rsq.rpart(biomarker.tree)

biomarker.tree

plot(biomarker.tree, uniform=T, branch=.4, compress=T,margin=0.055)

text(biomarker.tree, use.n=T, cex=0.5)

# prediction for individuals, results is the rate

# predict(biomarker.tree)[300]

rates = unique(predict(biomarker.tree))

rates

biomarker.tree2 <- prune(biomarker.tree, cp=cp)

biomarker.tree2

plot(biomarker.tree2, uniform=T, branch=.4, compress=T,margin=0.055)

text(biomarker.tree2, use.n=T, cex=0.7, all = TRUE)

CART_file= paste("CART diseasefree_allmarkers_minsplit",minsplit_now,".pdf")

pdf(file=CART_file)

plot(biomarker.tree, uniform=T, branch=.4, compress=T,margin=0.055)

text(biomarker.tree, use.n=T, cex=0.5)

dev.off()

CART_file= paste("CART diseasefree_allmarkers_pruned_minsplit",minsplit_now,".pdf")

pdf(file=CART_file)

plot(biomarker.tree2, uniform=T, branch=.4, compress=T,margin=0.055)

text(biomarker.tree2, use.n=T, cex=0.5)

dev.off()

### separate analysis for ACT – and ACT +

### ACT=no

data_ACT_no <- biomarker.df[which(biomarker.df$ACT==0), ]

minsplit_now=50

regform <- paste(

"Surv(data_ACT_no$DiseaseFree_survival, data_ACT_no$recurrence) ~",

paste(vars, collapse="+")

)

biomarker_ACT_no.tree <- rpart(as.formula(regform), data = data_ACT_no,

method = "exp",

control = rpart.control(minsplit = minsplit_now, cp = 0.01))

biomarker_ACT_no.tree$cptable

cp <- biomarker_ACT_no.tree$cptable[which.min(biomarker_ACT_no.tree$cptable[,"xerror"]),"CP"]

cp

plotcp(biomarker_ACT_no.tree)

rsq.rpart(biomarker_ACT_no.tree)

biomarker_ACT_no.tree

plot(biomarker_ACT_no.tree, uniform=T, branch=.4, compress=T,margin=0.055)

text(biomarker_ACT_no.tree, use.n=T, cex=0.5)

biomarker_ACT_no.tree2 <- prune(biomarker_ACT_no.tree, cp=cp)

biomarker_ACT_no.tree2

plot(biomarker_ACT_no.tree2, uniform=T, branch=.4, compress=T,margin=0.055)

text(biomarker_ACT_no.tree2, use.n=T, cex=0.7, all = TRUE)

CART_file= paste("CART diseasefree_allmarkers_ACT_no_minsplit",minsplit_now,".pdf")

pdf(file=CART_file)

plot(biomarker_ACT_no.tree, uniform=T, branch=.4, compress=T,margin=0.055)

text(biomarker_ACT_no.tree, use.n=T, cex=0.5)

dev.off()

CART_file= paste("CART diseasefree_allmarkers_pruned_ACT_no_minsplit",minsplit_now,".pdf")

pdf(file=CART_file)

plot(biomarker_ACT_no.tree2, uniform=T, branch=.4, compress=T,margin=0.055)

text(biomarker_ACT_no.tree2, use.n=T, cex=0.5)

dev.off()

# ACT = yes

data_ACT_yes <- biomarker.df[which(biomarker.df$ACT==1), ]

minsplit_now=50

regform <- paste(

"Surv(data_ACT_yes$DiseaseFree_survival, data_ACT_yes$recurrence) ~",

paste(vars, collapse="+")

)

biomarker_ACT_yes.tree <- rpart(as.formula(regform), data = data_ACT_yes,

method = "exp",

control = rpart.control(minsplit = minsplit_now, cp = 0.01))

biomarker_ACT_yes.tree$cptable

cp <- biomarker_ACT_yes.tree$cptable[which.min(biomarker_ACT_yes.tree$cptable[,"xerror"]),"CP"]

cp

plotcp(biomarker_ACT_yes.tree)

rsq.rpart(biomarker_ACT_yes.tree)

biomarker_ACT_yes.tree

plot(biomarker_ACT_yes.tree, uniform=T, branch=.4, compress=T,margin=0.055)

text(biomarker_ACT_yes.tree, use.n=T, cex=0.5)

biomarker_ACT_yes.tree2 <- prune(biomarker_ACT_yes.tree, cp=cp)

biomarker_ACT_yes.tree2

plot(biomarker_ACT_yes.tree2, uniform=T, branch=.4, compress=T,margin=0.055)

text(biomarker_ACT_yes.tree2, use.n=T, cex=0.7, all = TRUE)

CART_file= paste("CART diseasefree_allmarkers_ACT_yes_minsplit",minsplit_now,".pdf")

pdf(file=CART_file)

plot(biomarker_ACT_yes.tree, uniform=T, branch=.4, compress=T,margin=0.055)

text(biomarker_ACT_yes.tree, use.n=T, cex=0.5)

dev.off()

CART_file= paste("CART diseasefree_allmarkers_pruned_ACT_yes_minsplit",minsplit_now,".pdf")

pdf(file=CART_file)

plot(biomarker_ACT_yes.tree2, uniform=T, branch=.4, compress=T,margin=0.055)

text(biomarker_ACT_yes.tree2, use.n=T, cex=0.5)

dev.off()

#### Random forest ####

# random forest and variable importance whole sample

set.seed(415)

biomarker.df2 <- within(biomarker.df, rm(studynumber, diseasespecificsurvival,dead,followup))

View(biomarker.df2)

rsf1<- rfsrc(Surv(DiseaseFree_survival,recurrence) ~ ., data=biomarker.df2,ntree=1000)

rsf1.vimp <- vimp.rfsrc(rsf1)

plot(rsf1.vimp)

VIMP_figure= paste("Variable importance all patients.pdf")

pdf(file=VIMP_figure)

plot(rsf1.vimp,plots.one.page = FALSE)

dev.off()

# random forest and variable importance ACT_no

set.seed(415)

data_ACT_no2 <- within(data_ACT_no, rm(studynumber, diseasespecificsurvival,dead,followup))

rsf2<- rfsrc(Surv(DiseaseFree_survival,recurrence) ~ ., data=data_ACT_no2,ntree=1000)

rsf2.vimp <- vimp.rfsrc(rsf2)

plot(rsf2.vimp)

VIMP_figure2= paste("Variable importance NO ACT.pdf")

pdf(file=VIMP_figure2)

plot(rsf2.vimp,plots.one.page = FALSE)

dev.off()

# random forest and variable importance ACT_yes

set.seed(415)

data_ACT_yes2 <- within(data_ACT_yes, rm(studynumber, diseasespecificsurvival,dead,followup))

rsf3<- rfsrc(Surv(DiseaseFree_survival,recurrence) ~ ., data=data_ACT_yes2,ntree=1000)

rsf3.vimp <- vimp.rfsrc(rsf3)

plot(rsf3.vimp)

VIMP_figure3= paste("Variable importance ACT.pdf")

pdf(file=VIMP_figure3)

plot(rsf3.vimp,plots.one.page = FALSE)

dev.off()
