# Supplementary material for: KCNQ1 and lymphovascular invasion are key features in a prognostic classifier for stage II and III colon cancer
Source: BMC Cancer. 2022 Apr 8;22:372. doi: 10.1186/s12885-022-09473-9 (PMC8991490; doi:10.1186/s12885-022-09473-9)
Supplement: Supplementary file 4 — Additional file 4: Supplementary figure 1. Unpruned classification tree by CART analysis for ACT-untreated patients. Supplementary figure 2. Unpruned classification tree by CART analysis for ACT-treated patients. Supplementary figure 3. Variable importance graphs for both ACT-untreated and -treated patients. LVI = lymphovascular invasion / ITD = isolated tumour deposits / Emergency = emergency surgery / ACT = adjuvant chemotherapy. [file 12885_2022_9473_MOESM4_ESM.pptx]

## Slide 1
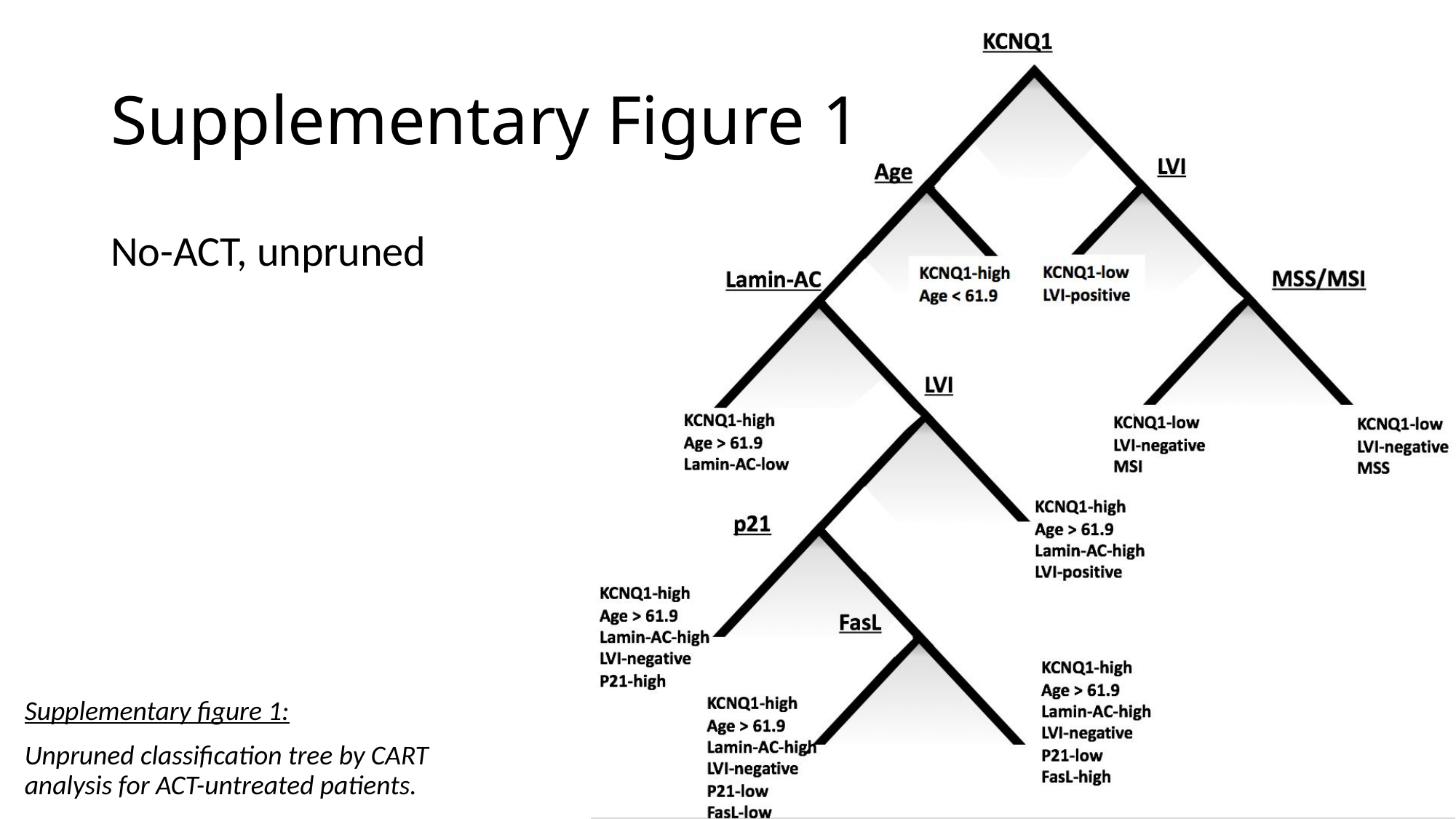

# Supplementary Figure 1
No-ACT, unpruned
Supplementary figure 1:
Unpruned classification tree by CART analysis for ACT-untreated patients.

## Slide 2
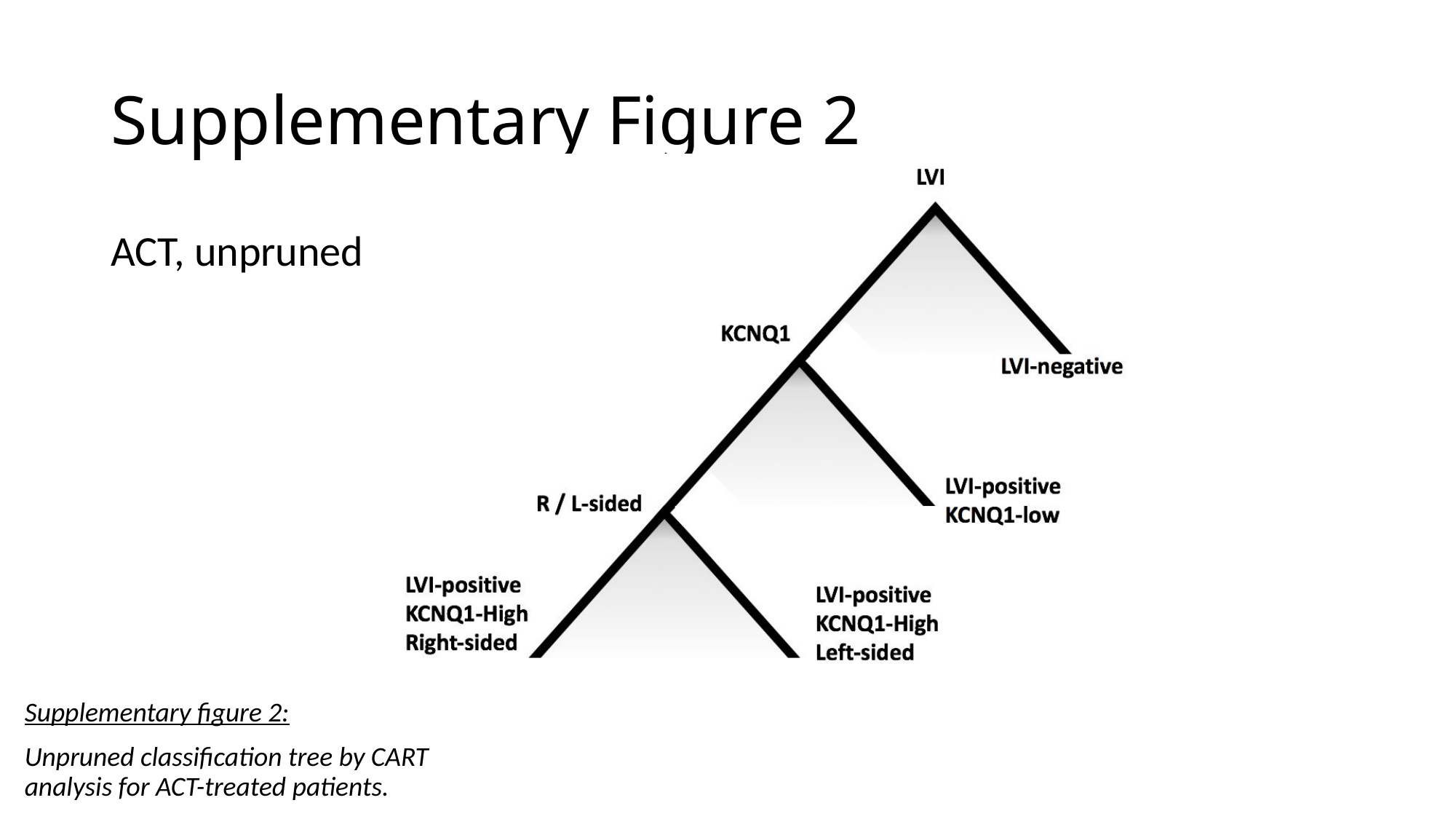

# Supplementary Figure 2
ACT, unpruned
Supplementary figure 2:
Unpruned classification tree by CART analysis for ACT-treated patients.

## Slide 3
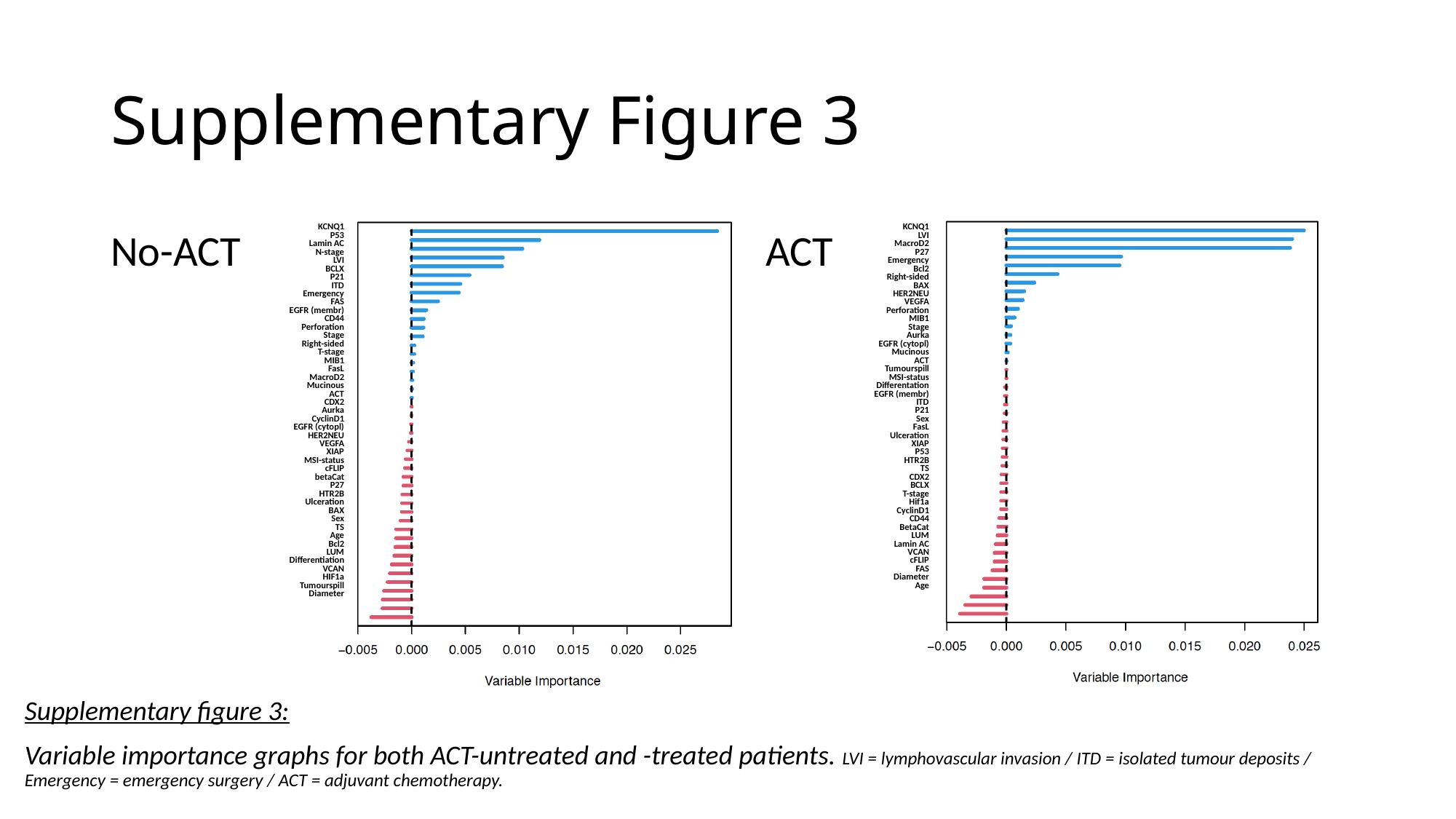

# Supplementary Figure 3
No-ACT					ACT
KCNQ1
P53
Lamin AC
N-stage
LVI
BCLX
P21
ITD
Emergency
FAS
EGFR (membr)
CD44
Perforation
Stage
Right-sided
T-stage
MIB1
FasL
MacroD2
Mucinous
ACT
CDX2
Aurka
CyclinD1
EGFR (cytopl)
HER2NEU
VEGFA
XIAP
MSI-status
cFLIP
betaCat
P27
HTR2B
Ulceration
BAX
Sex
TS
Age
Bcl2
LUM
Differentiation
VCAN
HIF1a
Tumourspill
Diameter
KCNQ1
LVI
MacroD2
P27
Emergency
Bcl2
Right-sided
BAX
HER2NEU
VEGFA
Perforation
MIB1
Stage
Aurka
EGFR (cytopl)
Mucinous
ACT
Tumourspill
MSI-status
Differentation
EGFR (membr)
ITD
P21
Sex
FasL
Ulceration
XIAP
P53
HTR2B
TS
CDX2
BCLX
T-stage
Hif1a
CyclinD1
CD44
BetaCat
LUM
Lamin AC
VCAN
cFLIP
FAS
Diameter
Age
Supplementary figure 3:
Variable importance graphs for both ACT-untreated and -treated patients. LVI = lymphovascular invasion / ITD = isolated tumour deposits / Emergency = emergency surgery / ACT = adjuvant chemotherapy.
